# Dissecting the Polygenic Basis of Cold Adaptation Using Genome-Wide Association of Traits and Environmental Data in Douglas-fir

**DOI:** 10.3390/genes12010110

**Published:** 2021-01-18

**Authors:** Amanda R. De La Torre, Benjamin Wilhite, Daniela Puiu, John Bradley St. Clair, Marc W. Crepeau, Steven L. Salzberg, Charles H. Langley, Brian Allen, David B. Neale

**Affiliations:** 1 School of Forestry, Northern Arizona University, 200 E. Pine Knoll, Flagstaff, AZ 86011, USA; bmw488@nau.edu; 2 Center for Computational Biology, Department of Biomedical Engineering, Computer Science and Biostatistics, John Hopkins University, 3100 Wyman Park Dr, Wyman Park Building, Room S220, Baltimore, MD 21211, USA; dpuiu@jhu.edu (D.P.); salzberg@jhu.edu (S.L.S.); 3 USDA Forest Service, Pacific Northwest Research Station, 3200 SW Jefferson Way, Corvallis, OR 97331, USA; brad.stclair@usda.gov; 4 Department of Evolution and Ecology, University of California-Davis, One Shields Avenue, Davis, CA 95616, USA; mcrepeau@ucdavis.edu (M.W.C.); chlangley@ucdavis.edu (C.H.L.); 5 Department of Plant Sciences, University of California-Davis, One Shields Avenue, Davis, CA 95616, USA; brallen@ucdavis.edu (B.A.); dbneale@ucdavis.edu (D.B.N.)

**Keywords:** cold adaptation, growth, phenology, cold hardiness, GWAS, GEA, Douglas-fir

## Abstract

Understanding the genomic and environmental basis of cold adaptation is key to understand how plants survive and adapt to different environmental conditions across their natural range. Univariate and multivariate genome-wide association (GWAS) and genotype-environment association (GEA) analyses were used to test associations among genome-wide SNPs obtained from whole-genome resequencing, measures of growth, phenology, emergence, cold hardiness, and range-wide environmental variation in coastal Douglas-fir (*Pseudotsuga menziesii*). Results suggest a complex genomic architecture of cold adaptation, in which traits are either highly polygenic or controlled by both large and small effect genes. Newly discovered associations for cold adaptation in Douglas-fir included 130 genes involved in many important biological functions such as primary and secondary metabolism, growth and reproductive development, transcription regulation, stress and signaling, and DNA processes. These genes were related to growth, phenology and cold hardiness and strongly depend on variation in environmental variables such degree days below 0c, precipitation, elevation and distance from the coast. This study is a step forward in our understanding of the complex interconnection between environment and genomics and their role in cold-associated trait variation in boreal tree species, providing a baseline for the species’ predictions under climate change.

## 1. Introduction

Understanding the fitness consequences of naturally occurring genetic variation is of great biological interest. In natural populations of widely distributed species, the challenge is to identify genes underlying traits that increase the species’ ability to survive, thrive and reproduce [1,2]. Species that encounter diverse ecological and environmental natural conditions may be subject to strong differential selection pressures across heterogeneous environments that counteract the homogenizing effects of gene flow and drift in the evolution of local adaptation [3,4,5]. In natural populations of forest tree species, the highly polygenic nature of climate and trait adaptation, the presence of phenotypic plasticity, and the complexity and scale of environmental variation across the species’ ranges present significant challenges for the study of phenotypic variation and local adaptation [6,7,8]. Therefore, a comprehensive understanding of the species’ potential for evolutionary change and adaptation to changing environments will require the study of genome-wide genetic variation across the species’ natural range and its association with ecologically important traits.

In the Northern hemisphere, the interactions between growth, phenology and cold hardiness are main determinants of survival in tree populations. Cold adaptation in forest trees involves significant physiological, cellular, genetic and morphological changes, and is highly synchronized with environmental cues such as photoperiod, day length and temperature [9,10]. Temperate and boreal forest tree species alternate periods of active growth and dormancy. Annual growth and tree development occur through a trade-off between maximizing growth under favorable environmental conditions to be able to compete for light, while avoiding cold injury from late spring frosts and early fall frosts [9,11]. Both bud burst (growth initiation) and bud set (growth cessation) are highly adaptive traits and parallel latitudinal or longitudinal environmental clines [11,12].

The identification of genes underlying variation in growth, phenology and cold hardiness has been particularly difficult due to the highly polygenic nature of the traits. Univariate genome-wide association studies (GWAS) successfully identify genes of major effect (in which a new advantageous mutation is rapidly driven to fixation) but have very low power to detect weakly selected loci, characteristic of polygenic adaptation [5,13,14]. Therefore, a multivariate GWAS will give a more accurate estimate of the number and effect sizes of genes contributing to phenotypic variation in polygenic traits. Adaptation to climatic or environmental variables also requires the study of many genes and interconnected environmental variables. In this case, multivariate genotype-environment association studies (GEA) are also more adequate than univariate methods. A comparison between univariate and multivariate methods will provide a good understanding of the different trait architectures in a species.

Douglas-fir (*P. menziesii*) is an economically and ecologically important species in western North America. Considered one of the most important sources of lumber worldwide, it is grown extensively in plantations throughout Europe [15] and is also a major source of timber in its natural range, particularly in the Pacific Northwest [16]. Douglas-fir’s wide geographic range spans a large gradient of environmental conditions and exhibits strong clines in relation to drought and cold tolerance [17,18,19]. Two varieties, *menziesii* and *glauca,* have been identified in coastal (Northern California—British Columbia) and intermountain regions (central Mexico—British Columbia); although phylogeographic studies using mitochondrial and chloroplast DNA suggest the presence of three, instead of two, distinct varieties (Coastal, Intermountain and Mexican) [20]. Common garden experiments focusing on climate adaptations of coastal Douglas-fir revealed that cold hardiness is highly correlated with cold-season temperature of the seed source [17], adaptations for coping with cold and drought overlap significantly [18,19], and that the species’ phenology likely lacks the ability to track climate change [21,22]. Cold adaptation in coastal Douglas-fir has also been studied through transcriptomic analysis [23], QTL mapping [24,25], and candidate gene based GWAS analysis [26]. Genome-wide studies of local adaptation were limited due to the absence, until recently, of a Douglas-fir reference genome [27]. Sequencing and annotating conifer genomes have been quite challenging due to their enormous genome sizes (>10 Gbp) and their high content of repetitive elements. Douglas-fir is a diploid species with 13 chromosomes and an estimated genome size of 16Gbp [27]. In this study, we combined genome-wide patterns of genetic variation obtained from whole-genome re-sequencing, phenotypic measurements of growth, phenology, emergence and cold hardiness, and environmental variables to understand the genomic basis of cold adaptation in coastal Douglas-fir. Our questions were: (1) What is the genomic architecture (number of genes, effect sizes, genomic and geographic location) of cold adaptation in the species? and (2) Which genes, pathways and biological processes are associated with variation in environments and traits related to cold adaptation in the species?

## 2. Materials and Methods

### 2.1. Whole-Genome Re-Sequencing

Seeds from ten individuals spanning most of the coastal Douglas-fir’s natural distribution (latitude 42–44 N, longitude 120–124 W degrees) were collected for whole-genome re-sequencing analysis. After collection, seeds were soaked in water at room temperature for four days, then haploid megagametophytes were dissected from each seed. DNA was extracted with a Qiagen DNeasy mini-prep Plant kit, and DNA quality and concentration were evaluated using nanodrop and picogreen on a Qubit 2.0 Fluorometer, respectively. Sequencing libraries were constructed using Illumina’s TruSeq Nano DNA Library Prep Kit [27]. Prior to amplification, DNA was fragmented (200 ng starting material and 550 bp target insert size), followed by end repair and size selection of fragments, adenylation of 3′ ends, and adapter ligation. PCR enrichment was performed in eight cycles. Barcoded libraries were combined into normalized pools and sequenced to >10 X coverage on an Illumina HiSeq 3000 using 150 bp paired-end reads at the University of California-Davis Genome Center.

### 2.2. SNP Calling

Raw reads from whole-genome re-sequencing data of ten Douglas-fir individuals were aligned to the 16 Gbp Douglas-fir reference genome version Psme v1.0 [27,28]; using Bowtie2 v2.2.9 [29]. The alignments were processed using SAMtools v1.3.1 and BEDtools v2.25.0; and SNPs were called using BCFtools v1.3.1 [30,31] with default parameters. A total of 458M SNPs were called. Filtering criteria included the removal of SNPs with a mapping quality < 20, depth of coverage < 8, and indels. All SNPs were given a score based on the sum of 16mer frequency sums of the 30 bp forward or reverse adjacent to the SNP. SNPs were discarded when the score was higher than 300. Moreover, SNPs were only called if present in scaffolds of 1kb or larger.

### 2.3. Sample Collection Prior to Genotyping

Seeds were obtained from 288 open-pollinated parent trees from throughout the range of Douglas-fir in western Washington and Oregon. Progeny from the parent trees were measured in a common garden study for adaptive traits including growth, phenology, emergence, and cold hardiness [32]. The same set of parents were used in previous association mapping studies [17,26,33]. Population stratification was based on geographic areas showing similar adaptive characteristics (defined as “provinces” in [17]), which roughly correspond with Douglas-fir breeding zones in Oregon and Washington.

### 2.4. DNA Extraction and SNP Genotyping

Seeds were soaked in a solution of 70% water and 30% of 3% hydrogen peroxide for 12 h. After that, megagametophyte haploid tissues from ten half-sib individuals for each family were pooled together to infer the maternal genotype. Megagametophytes DNA was extracted using the Qiagen DNeasy mini-prep Plant kit and an Eppendorf automated pipetting workstation. The extraction protocol included one day of tissue lysis and incubation at 96 °C, followed by several steps of precipitation and filtering. DNA quality and concentration were assessed using nanopore and picogreen on a Qubit 2.0 Fluorometer, respectively. Samples were genotyped using a custom-based multi-species Illumina Infinium SNP array comprising 80 k SNP markers from which 20 k were designed for Douglas-fir and 60 k for sugar pine. Ilumina’s Genome Studio Genotyping Module v2.0.4 was used to call genotypes, filter, and generate genotyping statistics for all samples and SNPs. Filtering was applied to Douglas-fir and sugar pine SNPs separately and the pass-filter SNP data were subsequently merged. All SNPs with a call frequency ≤ 0.65 and all individuals with a call rate ≤ 0.8 were not included. Further, markers were filtered out based on minor allele frequency (>0.01) to remove all monomorphic and low-quality SNPs. SNP functional annotations were obtained from aligning against the full NCBI non-redundant protein sequences database (nr) using BLASTP (*e*-value < 1 × 10^−10^), and by using the Douglas-fir’s genome annotations [34]. SNPs originally designed for sugar pine but that genotyped well in Douglas-fir were aligned to the Douglas-fir genome assembly with Minimap2 [35]. BEDtools v2.25.0 was used to assign SNPs to coding and non-coding regions of the Douglas-fir genome.

### 2.5. Population Structure

The number of genetic clusters was initially assessed with a principal component analysis (PCA) implemented in the “adegenet” R package [36,37]. Population clustering was further analyzed using the software fastSTRUCTURE [38]. Ten runs were completed for each value of K between one and ten. The chooseK script in fastSTRUCTURE was then used for each run to calculate which value of K was optimal. Because fastSTRUCTURE includes stochastic simulation, CLUMPP [39], implemented in the R package “POPhelper” [40] was used to combine the results of each run to optimize the number of clusters. Individuals were assigned to individual genetic clusters when *q*-value > 0.8; or to multiple clusters when *q*-value < 0.8. All R analyses were carried out on R version 3.5.3.

### 2.6. Isolation by Distance

To rule out the possibility that any patterns observed were due to isolation by distance (IBD), pairwise genetic and physical distances were calculated in the R package adegenet [36,37]. A mantel test was later performed to determine if there was significant IBD. Genetic distances were calculated as Nei’s distances [41] and fixation index (Fst) with the StAMPP package in R [42]. Geographic distances were calculated in base R, with the median latitude and longitude was used for the geographic location of populations. Mantel tests were carried out in ade4 R package [43].

### 2.7. Multivariate and Univariate Genome-Wide Association (GWAS) of Cold-Related Traits

Phenotypic data were measured as part of a large genecology experiment containing 1338 families from 1048 locations planted in a randomized plot design, seedlings were grown for two years in a common garden in Corvallis, Oregon [32]. A subset of those individuals was included in the study represented here. The full description of the methods used in the common garden can be found in [32]. In summary, seeds were collected from open-pollinated individuals in natural stands throughout the range of Douglas-fir in Oregon and Washington. Offspring of the maternal trees were grown for two years in a common garden located in Corvallis, Oregon. Tests were established over three years using different sets of families, with a common set of all three years to adjust for year effects as in [44]. Breeding values for all 23 growth, phenology, emergence, and cold hardiness traits (Appendix A, Table A1) were estimated by measuring the traits of seedlings being grown in the common garden. This study analyzed a small subset of this phenotypic data from 271 families.

A multivariate GWAS was performed by fitting a Bayesian sparse linear mixed model (BSLMM) in GEMMA v0.98 [45]. BSLMM uses Markov Chain Monte Carlo (MCMC) to associate markers and phenotypes by modeling all markers simultaneously while controlling for population structure and relatedness using the following model:

(1) y = 1 nμ + Xβ + u + e,

where 1n is an n-vector of 1s; μ is a scalar representing the phenotype’s mean; X is a matrix of genotypes from 271 families and 20,397 SNPs; β is the corresponding p-vector of the genetic SNP marker effects; u is an n-vector of random effects; and e is an n-vector of errors. Models were fitted for each of the 23 cold-related traits. In contrast to other multivariate GWAS methods, BLSMM captures the effect sizes of both small effect SNPs (α) and large effect SNPs (β). Therefore, PVE, the proportion of phenotypic variance explained by the combination of all small and large effect SNPs, reflects how well one could predict the phenotype from the available SNPs if β and u were known; and PGE reflects how well the phenotype is predicted by only using β [45]. Analysis was done with default options, which includes a burn-in of 100,000 steps, and 1,000,000 sampling steps. A posterior inclusion probability (PIP) > 0.01 (selected SNPs that had an effect in at least 1% of the models) was used as threshold for selection of associated SNPs [46,47].

In addition, a univariate GWAS using mixed linear model (MLM) was performed in TASSEL v.5. The first principal components of a PCA were used as co-variates to control for population structure, and a kinship matrix to account for relatedness [48]. Breeding values of progeny’s traits were determined as in [32]. The proportion of phenotypic variance explained by the SNP (R^2^), and the dominance and additive effects were also calculated with TASSEL v.5.

### 2.8. Multivariate and Univariate Genotype-Environment Association Analyses (GEA)

Elevation data were obtained from GIS coverages using a digital elevation model (DEM). Climate data were obtained using ClimateNA [49]. ClimateNA downscales PRISM data [50] to scale-free point data, allowing to more accurately predict maternal tree climate variables. All climate data are based off the averages for the years 1962–1990. Climate variables include monthly, seasonal and annual averages for minimum and maximum temperature, precipitation, daily temperature fluctuation and aridity (a ratio of precipitation to temperature); dates of 50 % probability of last spring frost and first autumn frost; frost-free period; and seasonal ranges in temperature and precipitation. All variables are detailed in Appendix A, Table A1. Correlations between all environmental (climatic and geographic) and phenotypic variables were investigated in R (version 3.6.1). Correlation plots were produced.

A redundancy analysis (RDA) as implemented in the vegan R package [51] was performed to estimate how much of the genetic variation was explained by environmental variables. RDA is a type of multiple regression that determines how much of the variation in one set of variables is explained by the variation in another set of variables. Environmental variables were investigated for correlation prior to carrying out the analysis. Variables were removed if their correlation coefficient was >0.8 with any other environmental variable. Since most variables were highly correlated with each other, the selected variables for the analysis were: Summer heat moisture index, distance to the sea, and precipitation as snow. Candidate SNPs were selected if falling outside of the 2.5 standard deviations of the mean loading score (*p*-value = 0.012) for each of the first two axes.

In addition to the multivariate GEA analysis, two univariate GEAs were performed using the programs Bayenv v.2 [52] and TASSEL v.5 [48]. Bayenv is based on a Bayesian method that control for effects caused by isolation-by-distance, population structure, and genomic background. The program estimates a covariance matrix of estimated allele frequencies to use as a null model of neutral genetic structure. This null model is then compared to a linear model between the allele frequencies and each environmental variable to test for improved fit over the null model. The software returns Bayes factors (BF) for each locus and, when using the nonparametric extension, also returns Spearman’s rank and Pearson’s correlation coefficients. Ten runs (100,000 iterations each) of the covariance matrix estimation were carried out and averaged together to ensure an accurate matrix across different runs. Loci were considered robust candidates for environmental selection if they had a BF ≥ 3 and were in the top 1% of Pearson’s correlation coefficient. TASSEL univariate analyses were performed using each environmental variable as the vector of observations, and the SNP markers, population structure and kinship as fixed effects in an MLM association analysis. The first three principal components of a PCA of environmental variables were also tested for associations with the SNP markers.

### 2.9. Genome Scan for Detection of Selection Signatures

Pcadapt R package [53] was used to detect signatures of selection in the 20.3 k SNP set. Pcadapt estimates principal components (K) using a principal component analysis; and later calculates the correlation between genetic variation and the first K principal components using Mahalanobis distance to detect outliers [53]. A q-value threshold of 0.1 was used. Pcadapt was chosen over other genome scans methods because it does not require grouping individuals into a-priori defined populations and it is not sensitive to admixed individuals in the dataset.

## 3. Results

### 3.1. SNP Calling and SNP Genotyping

**Figure A1:**
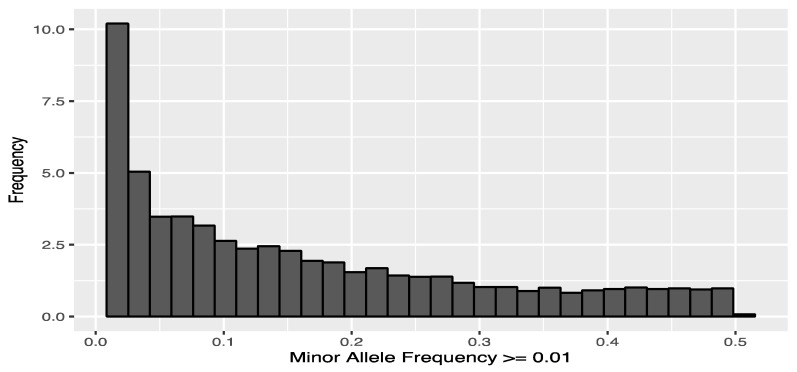
Histogram showing the distribution of minor allele frequency for the 20,397 SNPs included in this study.

Of the 458M SNPs called in the whole genome re-sequenced individuals, a total of 1.2M SNPs were retained after filtering following the SNP calling process. All SNPs were scored by Illumina using in silico design scores to maximize the number of markers for genotyping that will provide high conversion rates. As a result, 20 k best-scoring SNPs were included in the Illumina Infinium array for SNP genotyping. From this, genotypes were obtained from 16,146 SNPs. Posterior genotype clustering, and removal of monomorphic and low-quality SNPs led to 20,397 SNPs, from which 5815 were originally designed for sugar pine. SNPs selected for posterior analyses were distributed along 5892 scaffolds in the Douglas-fir genome and matched 6833 coding regions (4301 genes plus 2532 transcripts from unknown genes). The distribution of SNPs minor allele frequencies can be seen in Appendix A, Figure A1.

### 3.2. Population Structure

**Figure 1:**
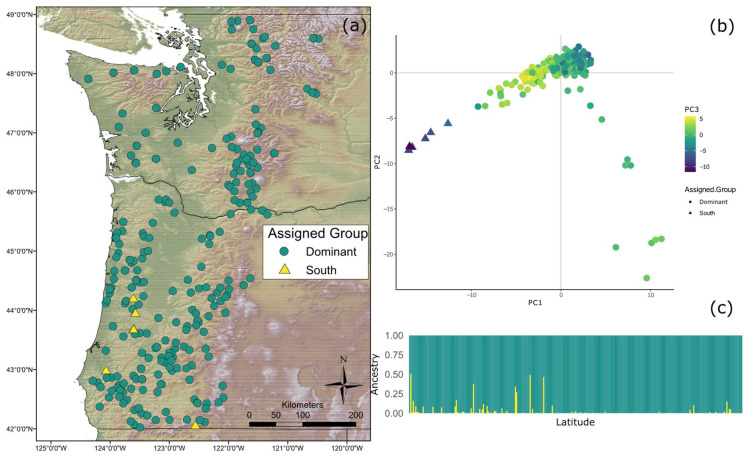
(**a**) Map of sample locations by population structure. Green circles represent Coastal Dominant (CD) individuals while yellow triangles represent individuals with significant Coastal South (CS) ancestry; (**b**) Population structure based on principal component analysis (PCA) results. PCA was carried out in Adegenet and only accounts for genotype. The first three principal components are represented by the *x*-axis, *y*-axis, and the color, respectively; (**c**) Structure plot of sampled individuals as determined by fastSTRUCTURE. Each bar along the *x*-axis represents an individual and the individuals are ordered by latitude (increasing left to right). Green represents ancestry from the CD genetic group, while yellow represents the level of ancestry from the CS genetic group.

A K value of 2 was shown to be most optimal using the choose K script included in fast STRUCTURE. Distruct [54] used the CLUMPP output of all 10 runs where K = 2 to make an ancestry plot of each individual (Figure 1). Results of the fastSTRUCTURE analysis showed that two distinct genetic clusters exist within the study zone; a dominant type throughout the sampled range up to the Canadian border (“Coastal Dominant”), and a smaller one existing in Southern Oregon (“Coastal South”). Individuals with mixed ancestry from both clusters were found in Southern Oregon. The map resulting from assigning individuals to the distinct genetic clusters can be seen in Figure 1. The results of the principal component analysis also showed two fairly distinct groups (Figure 1).

### 3.3. Correlations among Cold-Related Phenotypic Traits and Environmental Variables

**Figure A2:**
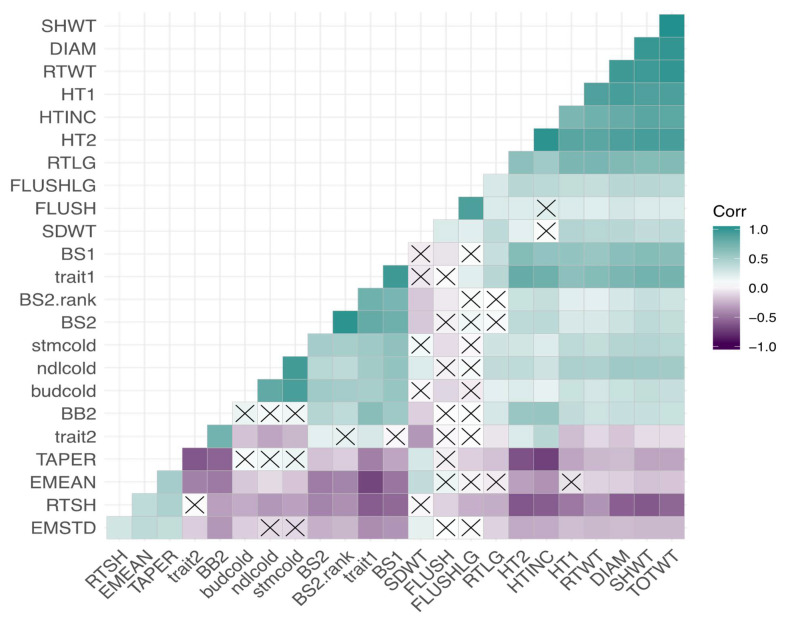
Correlations among phenotypes. A heatmap is shown with a gradient between purple (negative correlation coefficient) and teal (positive correlation coefficient) with white representing a correlation coefficient of zero. No significant correlations are denoted by an “×” in each box. Phenotypes’ IDs can be found in Table A1.

**Figure A3:**
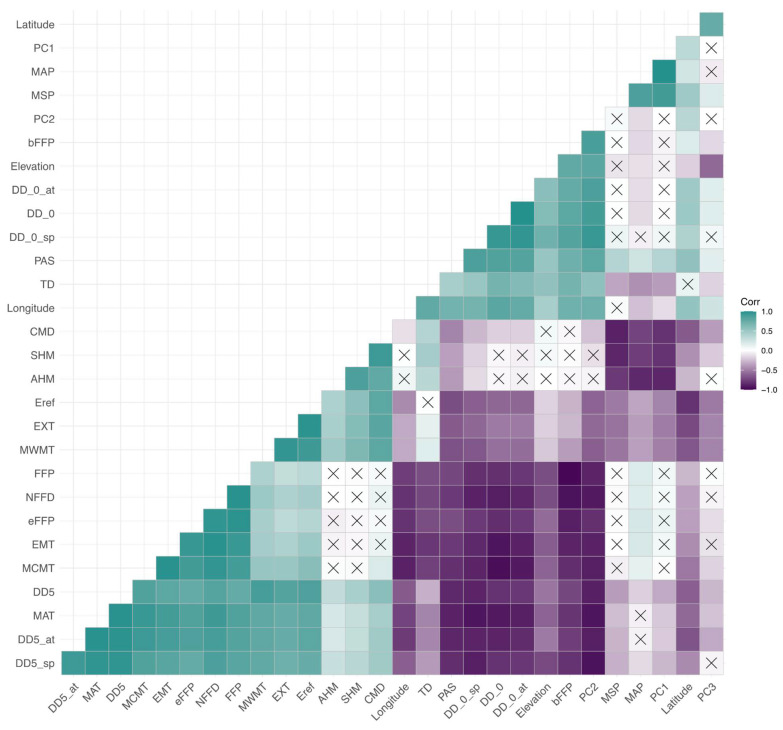
Correlations among environmental variables. A heatmap is shown with a gradient between purple (negative correlation coefficient) and teal (positive correlation coefficient) with white representing a correlation coefficient of zero. No significant correlations are denoted by an “×” in each box. Environmental variables’ IDs can be found in Table A2.

**Figure A4:**
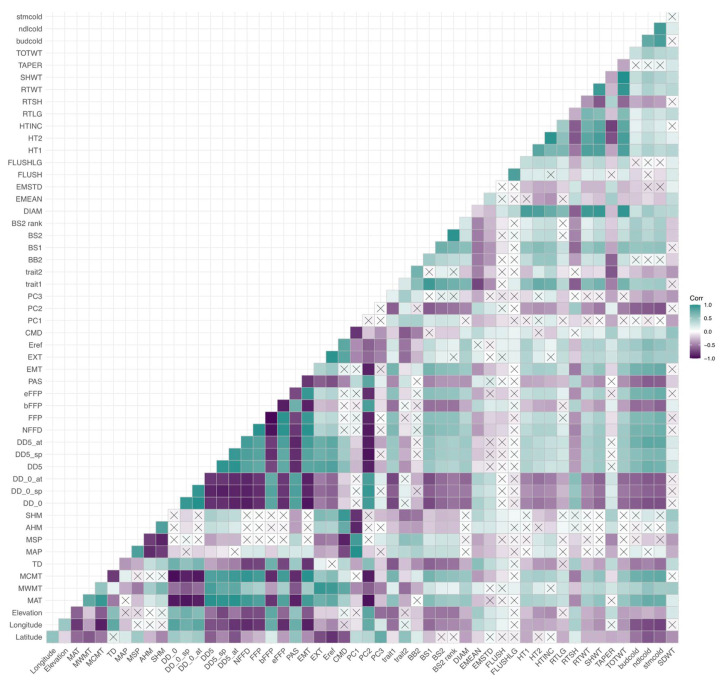
Correlations among phenotypes and environmental variables. A heatmap is shown with a gradient between purple (negative correlation coefficient) and teal (positive correlation coefficient) with white representing a correlation coefficient of zero. No significant correlations are denoted by an “×” in each box. Phenotypes’ IDs can be found in Table A1, and environmental variables’ IDs can be found in Table A2.

All correlations among phenotypic traits and environmental variables are represented in heatmaps in Appendix A, Figure A2, Figure A3 and Figure A4. The three measures of cold damage (percentage of damaged tissue in needle, stem, and bud tissue) are positively correlated with each other as well as with growth rate (both height measurements, height increment, root, shoot, and total weight, and root length). These measures of cold hardiness had weak negative correlations with measures of emergence and root to shoot ratio. Most phenotypes were significantly correlated with each other, with the exception of the propensity to second flush and length of second flush, which were correlated with each other, but not strongly correlated to any other variables (Appendix A, Figure A2). Correlations within environmental variables can largely be broken into two groups, one containing all temperature variables and continentality; with a second group consisting of precipitation variables, PCs 1–3 and geographic variables (Appendix A, Figure A3). Trait 1 was negatively correlated with elevation, longitude, and continentality (TD) and positively correlated with Mean Annual Temperature (MAT) and Mean Coldest Month Temperature (MCMT). Trait 2 was negatively correlated with Climate Moisture Deficit (CMD), Summer heat moisture (SHM), and positively correlated with latitude (Appendix A, Figure A4).

### 3.4. Genome Scan for Detection of Selection Signatures

**Figure 2:**
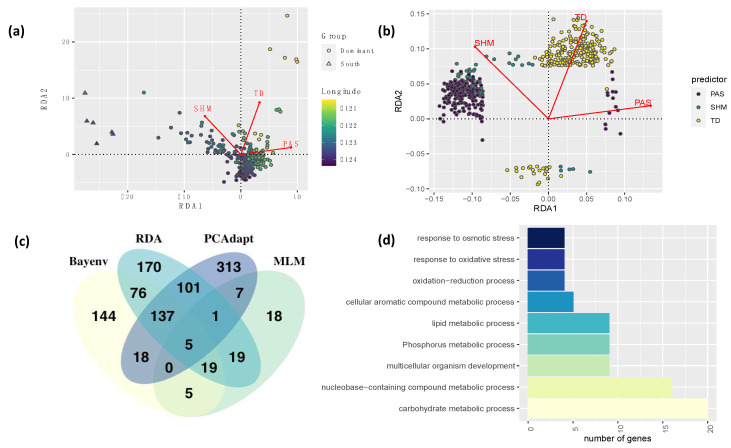
Results of the Genotype-Environment association (GEA) analyses. Results of the multivariate RDA analysis by (**a**) individuals along longitudinal gradients (color scale), and (**b**) SNP markers for significant environmental variables such as Precipitation as snow (PAS), Summer Heat Moisture Index (SHM), and continentality (TD) based on 20.3 k genome-wide SNP markers identified for coastal Douglas-fir; (**c**) Venn diagram of combined results of all GEA methods; and (**d**) Main gene ontologies of genes identified in all GEA methods.

PCAdapt identified 582 outlier loci distributed across 290 scaffolds in the Douglas-fir genome (Supplementary Material Table S1). From those SNPs, 225 were from coding regions (156 matched Douglas-fir genes and 69 matched transcripts with unknown genes), and the remaining SNPs were from non-coding or unidentified genomic regions. From the 582 outlier loci, 271 were significant in GWAS analyses and 269 in GEA results (Figure 2).

### 3.5. Isolation by Distance

**Figure A5:**
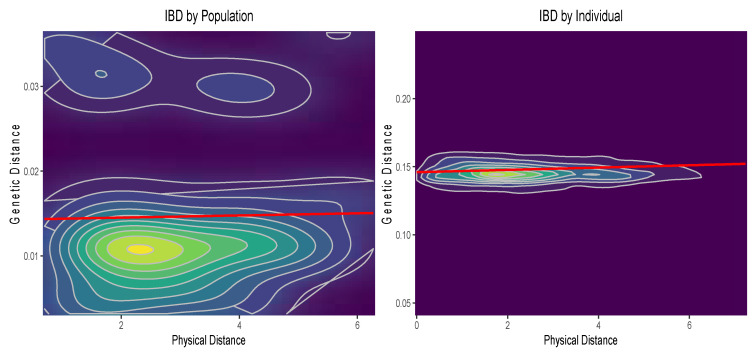
Results of the Mantel tests. IBD refers to Isolation by distance, and “physical distance” refers to geographic distance among populations.

When individuals were not grouped into populations, mantel tests revealed very low levels of isolation by distance (*r* = 0.09, *p*-value = 0.001). There was no significant relationship between genetic distance and geographic distance when individuals were grouped into populations (*r* = 0.02, *p*-value = 0.37). See Appendix A, Figure A5 for a plot of the results. This, combined with the results of fastSTRUCTURE, indicated a weak population structure in the dataset.

### 3.6. Univariate and Multivariate Genome-Wide Association Study of Cold-Related Traits

The MLM model implemented in TASSEL identified 799 significant associations after a false discovery rate (fdr) correction for multiple-testing [55]. A total of 690 associations were significant for additive effects (Supplementary Material Table S2). Associations were found among 237 SNPs and 20 traits. Of the 237 SNPs, 170 matched coding regions (126 genes and 44 transcripts) and the others were non-coding (Supplementary Material Table S2). Minor allele frequencies of significant SNPs ranged from 0.01 to 0.48 (mean = 0.04, standard deviation = 0.06). Trait 1 (a linear combination of several growth traits), for which higher values represent higher vigor (faster growth, later budset, earlier emergence, greater partitioning to shoot vs. root) was associated with 106 SNPs, and root to shoot ratio (RTSH) was associated with 115 SNPs based on TASSEL results. The number of SNPs associated to each trait can be found in Table 1.

**Figure A6:**
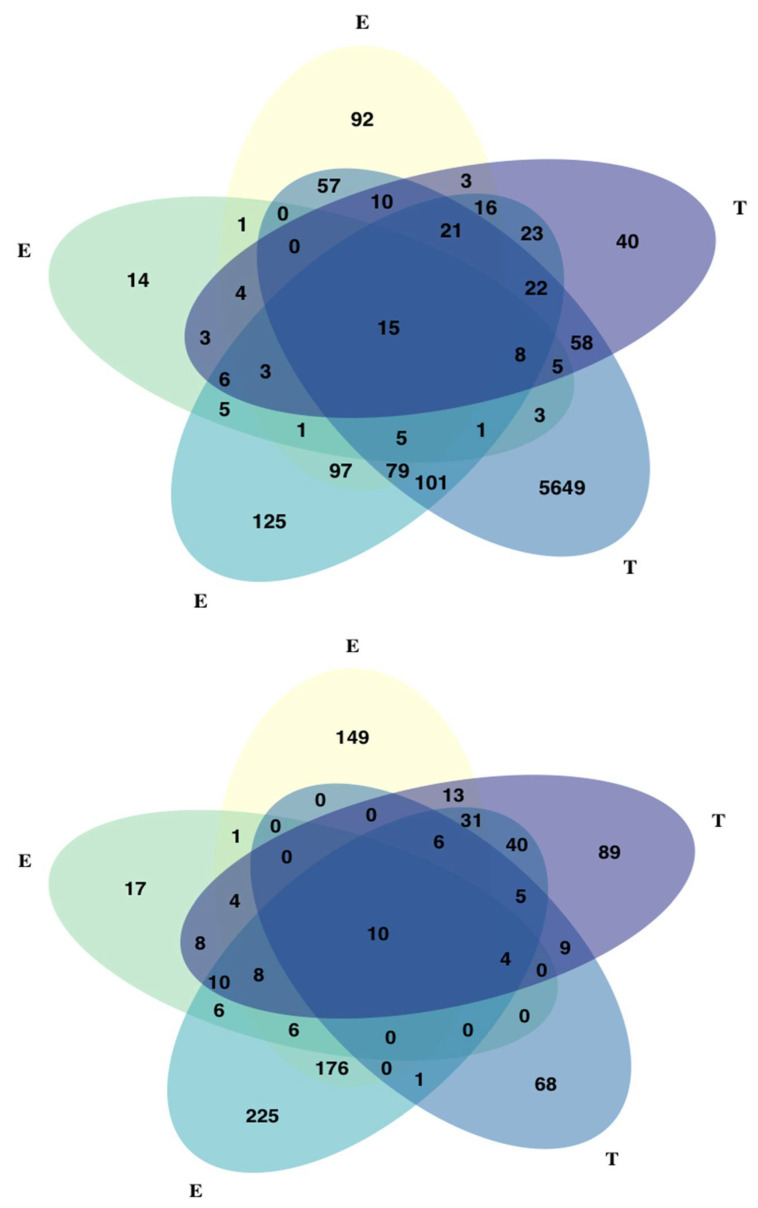
Combined results of all GEA and GWAS analyses. GWAS analyses (“T”) are Mixed Linear Model (MLM) in purple, and Bayesian Sparse Linear Mixed Model (BSLMM) in blue. GEA analyses (“E”) are Bayenv in yellow; RDA in dark green and MLM in light green. In the graph on top, BSLMM PIP threshold for significant SNPs was 0.01, in the graph at the bottom, PIP threshold was 0.05. The number and identity of shared SNPs among all methods (dark blue) is almost consistent between the two graphs.

In contrast, GEMMA identified 7536 significant associations among 6034 SNPs and all 23 traits (Table 1, Supplementary Material Table S3). Significant SNPs were matched to 2173 Douglas-fir genes and 1117 transcripts (from unknown genes). Minor allele frequencies of significant SNPs ranged from 0.01 to 0.5 (mean = 0.15, standard deviation = 0.13). Traits with large (>1000) number of associated SNPs were needle cold damage, bud cold damage and Trait 2. Increasing the threshold of posterior inclusion probability (PIP) from 0.01 to 0.05 and 0.1 significantly reduced the number of SNPs associated with needle cold damage, bud cold damage and Trait 2, but had little effect on other traits. The number of shared SNPs found across all GWAS and GEA analyses also remain relatively unchanged when changing the PIP threshold (Appendix A, Figure A6).

Individual SNP markers explained from 6 to 24% (mean = 9.9, stdev = 3.1) of the phenotypic variation for all traits, whereas combined SNP markers explained up to 93% (Table 1, Supplementary Material Table S2). Cold hardiness traits (needle and stem cold damage) had high PVE but low PGE (proportion of genetic variance explained by sparse effects), suggesting that these traits are highly polygenic and mainly controlled by large numbers of genes of small effect. This was further supported by the fact that traits exhibiting this pattern had very few SNPs identified by univariate models (Table 1), but many SNPs identified by GEMMA. Both large and small effects genes seem to be important controlling growth, emergence and phenology traits. None of the studied traits seemed to be only controlled by large effect genes.

### 3.7. Univariate and Multivariate Genotype-Environment Association Analyses (GEA)

**Figure A7:**
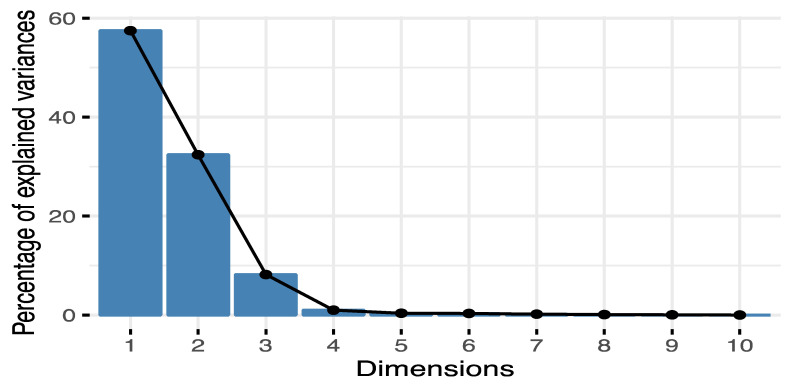
Proportion of the variance explained by the principal components of the PCA of environmental variables. Each bar represents % variance explained by a principal component. Most of the variance is explained by the first two principal components.

**Figure A8:**
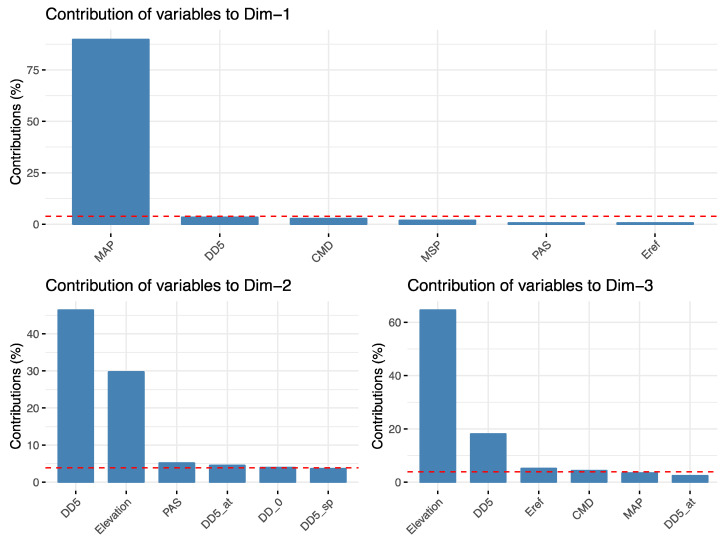
Environmental variables represented by the first two principal components of the PCA of environmental variables. Each pane shows a different principal component. Within each pane, each bar represents the proportion each variable contributes to each axis. The first axis is primarily annual precipitation and winter precipitation. The second axis primarily represents elevation.

TASSEL univariate association analyses identified 74 SNPs significantly associated with 17 environmental variables (Supplementary Material Table S4). Most SNPs were related to cold temperature variables, with 55, 28, and 16 SNPs associated with Degree days below 0 in autumn, year-wide, and spring (DD_0_at, DD_0, DD_0_sp), respectively. Results of the PCA of environmental variables indicated that principal component 1 explains 57.5% of the variation in the data and is almost entirely represented by mean annual precipitation (MAP), whereas PC2 explains 32.4% and is represented by growing degree days (DD_5) and elevation (Appendix A, Figure A7 and Figure A8). Bayenv identified associations among 404 SNPs and 22 environmental variables. These SNPs matched 110 genes and 43 transcripts from unknown genes (Supplementary Material Table S5). Traits were associated with 1 to 77 SNPs each. Minor allele frequencies of significant SNPs had a mean of 0.04 and a standard deviation of 0.03. From the 404 SNPs, 260 were also significant in other GEA analyses (Figure 2).

The multivariate (RDA) GEA method identified a total of 528 significant SNPs for continentality (255 SNPs), precipitation as snow (219 SNPs), and summer heat moisture index (54 SNPs) (Supplementary Material Table S6). Of these 528 SNPs, 144 matched genes and 58 transcripts (from unknown genes). Each of these variables were correlated to other environmental variables, suggesting the combined effect of groups of individual variables associated with an aspect of climate (Appendix A, Figure A3). Figure 2 shows the individuals and SNPs plotted in the ordination space. Individuals of genetic cluster coastal south (southern Oregon, see Figure 1) grouped together and separate well from the rest of the individuals mostly on summer heat moisture index. Individuals from this cluster also tend to be at lower latitudes and longitudes than the rest of the population. Minor allele frequencies had a mean of 0.07 and a standard deviation of 0.07. Main gene ontologies of combined results across all GEA methods were metabolic processes (carbohydrate, lipid, nucleobase, phosphorus, and cellular aromatic), response to stress (oxidative and osmotic), and developmental processes involved in reproduction (Figure 2). When comparing the results of all methods, 385 SNPs (130 genes and 54 transcripts from unknown genes) were significant in at least one GEA and one GWAS analysis (Table 2). From them, 15 SNPs were significant in all univariate and multivariate GEA and GWAS analyses (Supplementary Material Table S7, Appendix A, Figure A6).

## 4. Discussion

In agreement with previous studies of natural populations of highly-outcrossing, widely distributed boreal and temperate conifer species [4,6,9,26,33], we found strong evidence for cold adaptation despite low population structure and (potentially) high gene flow. Our Bayesian clustering and PCA analyses revealed very little population structure within the study area, with gene flow mainly occurring asymmetrically from northern to southern populations, and from high to low elevations. Steep gradients of selection are likely balancing large rates of migration (via pollen) to shape steep elevational clines. These clines are most likely produced by large differences in the frequency of alleles that are either common (and widespread) or rare (but frequent at high elevations). Previous studies have shown strong evidence of steep phenotypic and environmental clines within the coastal variety [17,18,19,32,56]. Significant divergence in allele frequency was also found in genome-wide studies comparing high and low elevation populations of *Pinus yunnanensis* [57]. In contrast, studies in this and other forest tree species suggested small to moderate shifts in allele frequency of adaptive alleles [6,58,59], and the presence of intermediate-frequency polymorphisms characteristic of recurrent selective sweeps [60]. Further studies are required to know whether recurrent selective sweeps, demographic processes and/or introgression from the interior (mountainous) variety explain the pattern observed.

Our results highlight the role of both coding and non-coding regions in cold adaptation of the species. We believe these regions are widely distributed across the genome, however, in the absence of a high-density linkage map or a chromosome-scale reference genome, our study could only identify genomic locations at the scaffold-level. We identified multiple SNPs or genes within the same scaffolds. Due to the highly fragmented nature of the Douglas-fir reference genome, we assume genes located in the same scaffold have a high chance of being linked. A widespread genomic location of adaptive genes was also found in recent GWAS and GEA analyses in *Pinus taeda* [6,61], *Pinus lambertiana* [62] and *Populus balsamifera* [63].

### 4.1. Polygenic Basis of Cold Adaptation in Coastal Douglas-fir

Both GWAS and GEA analyses suggested a polygenic basis of cold adaptation, with many genes associated with cold-related traits and climate adaptation. This is coincident with recent genome-wide analyses in widespread, outcrossing plant species with large population sizes such as *Zea mays* [64], *Populus trichocarpa* [65], *Pinus contorta* [66], and *Pinus sylvestris* [67]. Trait architecture in coastal Douglas-fir seems to be more complex than previously suggested, with traits (such as growth, emergence and phenology) controlled by both large and small effect genes, and others (cold hardiness) mainly controlled by large numbers of small effect genes. None of the traits was only controlled by major effect genes. The main differences between univariate and multivariate GWAS results were found in cold hardiness and trait2. While univariate MLM identified less than a handful of SNPs, multivariate BSLMM identified 1 or 2 thousand SNPs associated with each trait. Most of these SNPs had very small effect sizes, and even BSLMM failed to capture them when the posterior inclusion probability (PIP) threshold increased from 0.01 to 0.05. While the first results (PIP > 0.01) might overestimate the number of associated SNPs by incorporating false positives, the second results (PIP > 0.05) fail to capture SNPs of very small effect significantly increasing the number of false negatives. Another important difference between univariate and multivariate GWAS results is the percentage of phenotypic variance explained by SNPs. Individual SNP markers explained from 6 to 24% of the phenotypic variation for all traits, whereas combined SNP markers explained up to 93% (Table 1, Supplementary Material Table S2).

Aspects of climate are highly correlated with each other, complicating the interpretation of genome-wide environmental associations. Our GEA analyses identified several hundreds of SNPs associated with a large number of temperature and precipitation related variables (Supplementary Material Tables S4–S6), suggesting the study of climate adaptation requires the analysis of groups instead of individual environmental variables. Previous studies have identified modular aspects of climate adaptation in species such *Pinus contorta* [68] and *Pinus taeda* [6]. Multivariate methods identified more SNPs but the difference between univariate and multivariate GEA methods was more subtle than between GWAS methods, with hundreds of significant SNPs shared across methods (Figure 2). Univariate Bayenv and multivariate RDA identified the same 237 significant SNPs, but univariate MLM only found 6 common significant SNPs with multivariate RDA and 29 with Bayenv (Figure 2). Pcadapt, the outlier method, identified 383 SNPs in common with other GEA methods (Figure 2). When comparing the results of all GEA and GWAS methods, 385 SNPs (130 genes and 54 transcripts from unknown genes) were significant in at least one GEA and one GWAS analysis. These genes indicate a complex genomic architecture of cold adaptation in the species with many genes involved in many important biological functions related to growth, phenology and cold hardiness and that strongly depend on variation in environmental variables such Degree days below 0c, amount of precipitation, elevation and distance from the coast (TD) (Table 2).

### 4.2. Trade-Offs between Growth and Cold Hardiness

Trees living in cold environments must balance the timing of growth initiation with the risk of frost [69]. If a tree initiates growth too early, before the last spring frost, it risks tissue damage or death. On the other hand, if a tree initiates growth too late, it loses out on potential growing season and might not be able to compete for light with nearby trees. This trade-off between growth and cold hardiness have been observed in several temperate and boreal tree species, including *Pseudotsuga menziessii* [9,26], *Picea sitchensis* [70], *Populus trichocarpa* [71], *Pinus sylvestris* [67,72,73], *Abies sachalinensis* [74], *Acer rubrum*, *Betula alleghaniensis*, *Quercus rubra* and *Juglans nigra* [75].

**Figure 3:**
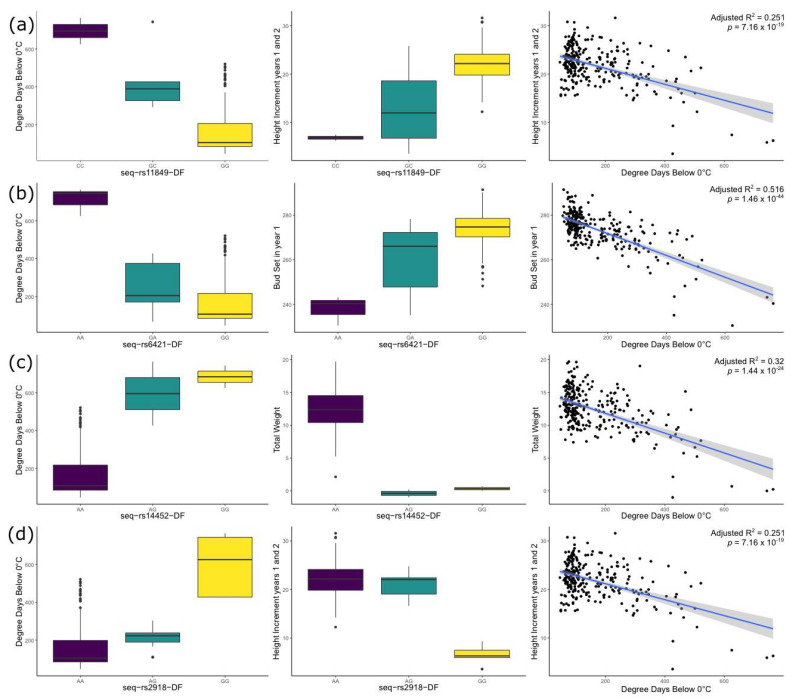
SNPs showing significant associations with cold-related traits and environmental variables in all univariate and multivariate GWAS and GEA analyses. (**a**) SNP seq-rs11849-DF (gene PSME_39361, 1-deoxy-D-xylulose 5-phosphate reductoisomerase protein, terpene backbone biosynthesis pathway) is associated with the first canonical trait, BS1, DIAM, HT1, HT2, HTINC, RTLG, TOTWT and SHWT; and environmental variables such as continentality, Degree days below 0c and Precipitation as snow; (**b**) SNP seq-rs6421-DF (gene PSME_0092, ubiquitin-40S ribosomal protein S27a, Ubiquitin mediated proteolisis) is associated with the first and second canonical traits, BS1, DIAM, HT2, HTINC, RTSH; and environmental variables such as continentality, Degree days below 0c, Precipitation as snow and Mean Summer Precipitation; (**c**) SNP seq-rs14452-DF (gene PSME_51103, protein CASP isoform X2, transcription regulation) is associated with BS1, DIAM, HT1, HT2, HTINC, RTLG, RTSH, RTWT, SHWT, TOTWT, trait1; and several environmental variables such as continentality, Degree days below 0c and Latitude.; (**d**) SNP seq-rs2918-DF (gene PSME_27219, cyclin-dependent kinase C-2-like, cell cycle control) is associated with all growth and emergence traits, BB2, BS1 and needle cold hardiness; and environmental variables such as continentality, and Degree days below 0c and PC1. Keys to traits and environmental variables can be found in Appendix A, Table A1 and Table A2.

Our results showed the complex relationships between genotypes, growth (DIAM, HT1, HT2, HTINC), emergence (EMEAN, EMSTD), phenology (BS1, BS2, BB2), cold hardiness, and environmental variables such as degree days below 0c, precipitation as snow, and continentality (TD) (Figure 3). The importance of these environmental variables for cold adaptation was suggested in previous common garden studies in the species [18,19,26,32]. As we move further away from the sea towards the mountains (decreasing longitude and increasing continentality), the weather gets colder with a higher number of degree days below 0c, and increased snow precipitation, characteristic of longer winter seasons. Longer winter seasons bring shorter growing seasons, in which Douglas-fir develops new growth earlier but also stops growing earlier than their coastal counterparts. As a consequence of this, general growth (height and diameter) is reduced but cold hardiness is increased at lower longitudes and higher elevations. For example, 106 SNPs showed associations with Trait 1 (Table 1), a linear combination of several growth traits. Trait 1 inversely correlates with longitude, elevation, continentality and winter temperatures suggesting that higher values of Trait 1 are correlated with higher vigor (faster growth, later budset, earlier emergence, greater partitioning to shoot vs. root), which is coincident with previous common garden studies suggesting an association between Trait 1 and lower drought and cold tolerance [18,32]. Most of the genes associated with both traits and environmental variables also showed evidence of trade-offs between growth and cold hardiness (Table 2).

### 4.3. Functional Characterization of Genes Associated with Cold Adaptation

Cold adaptation in forest trees involves significant physiological, cellular, genetic and morphological changes [9,10]. In our study, we found that genes associated with cold adaptation affect many important biological processes such as primary (carbohydrate, lipid, aminoacid and RNA) metabolism, secondary metabolism (terpenes, steroids, vitamins and chlorophyll), growth and reproductive development, transcription regulation, stress and signal transduction, and DNA processes (Table 2). Both carbohydrate and lipid metabolism are deeply modified during cold stress in temperate and boreal trees. Carbohydrate metabolism changes include the accumulation of non-reducing disaccharides (sucrose and raffinose) and the increase in polysaccharide (starch) breakdown, which produces more energy to compensate for reduced photosynthetic activity during freezing stress. In our study, we found genes involved in cell wall pectin (gene PSME_35569); starch and sucrose metabolism (gene PSME_01956); fructose and mannose metabolism (gene PSME_39947); pentose phosphate pathway (PSME_32494); glycolysis (PSME_39868 and PSME_34754); sugar transport (PSME_46816 and PSME_47335) and nine other genes involved in similar processes. Most of these genes were associated with cold hardiness traits (ndlcold and budcold), and environmental variables such as Continentality (TD) and others (Table 2). Similar to carbohydrates, membrane lipids adjust their composition to protect cell walls against dehydration-related freezing injury [10,11,76]. In our study, we only found one gene involved in the metabolism of glycerophospholipids (PSME_32128), which was correlated with mean coldest month temperature (MCMT) and trait2 (Table 2). Genes involved in arginine, proline, β-alanine, cysteine and methionine metabolism were also found in this study (PSME_23694, PSME_30851 and PSME_40791).

Plant secondary metabolites play an important role in adaptation to changes in the environment [77]. In our study, we found four SNPs involved in terpenoid biosynthesis (3 of them matching the same gene PSME_39361). One of those SNPs, seq-rs11849-DF (1-deoxy-D-xylulose 5-phosphate reductoisomerase) produces a non-synonymous change and was found to be significantly associated in all five GEA and GWAS analyses. The homozygote form containing the minor allele (CC) is present at eastern longitudes and higher elevations (usually > 900 masl) and is associated with slower growth and earlier bud set (Table 2, Figure 3). Other important genes involved in terpene biosynthesis (specifically diterpenes) are members of the Cytochrome P450 super gene family [78]. We found Cytochrome P450 gene PSME_03065, from which the homozygote form containing the minor allele (GG) is present at higher elevations (usually > 1000 masl) and is associated with lower values of trait 2 (later bud burst and lower partitioning to diameter vs. height) and precipitation as snow higher than 400 mm.

Plants employ complex signaling pathways to regulate the expression of defense and stress genes and other mechanisms that allow resistance to environmental stress [79]. The ubiquitin-proteasome system controls the degradation of most proteins in the cells. It provides a rapid strategy to control many cellular processes by degrading specific proteins, playing a critical role in the regulation of many biological processes such as hormonal signaling, growth, embryogenesis, senescence and environmental stress [80,81]. The initiation of ubiquitination (protein degradation) requires an E1 enzyme joining a Ubiquitin protein and follows with a three-step conjugation cascade (E1 > E2 > E3) that detects specific ubiquination signals [81]. Our study identified a strong candidate for that first ubiquitin protein in the ubiquitination process in Douglas-fir (SNP seq-rs6421-DF, Gene PSME_00292 ubiquitin-40S ribosomal protein S27a). All univariate and multivariate methods in this study identified this SNP as significantly associated with several traits and environments. The homozygote form containing the minor allele (AA) is present at eastern longitudes and higher elevations (usually > 900 masl) and is associated with earlier bud set and slower growth (Table 2, Figure 3). Cold stress induces the degradation of transcription factor ICE1, process that is mediated by a RING-type E3 ligase. In *Arabidopsis*, overexpression of HOS1 (RING-type E3 ligase) and AtCHIP (U-box type E3 ligase) led to reduced cold hardiness [81]. In our study, we have found two U-box genes (PSME_03290 and PSME_37900) and one RING-type E3 gene (PSME_02072) associated with several growth traits and continentality (TD) in Douglas-fir.

Members of the thaumatin family (“antifreeze proteins”) are upregulated during cold stress and are potentially regulated by photoperiod in *P. sitchensis* [11]. In our study, we found thaumatin gene PSME_47504, which is associated with several growth traits, degree-days below 0c and continentality. Peroxidases (such as genes PSME_37207 and PSME_46413) are upregulated during cold stress to protect against oxidative damage caused by an increase in reactive oxygen species [11,82]. Members of the ABC super gene family were found to be key players in defense mechanisms against different herbivores [83,84] and pathogens [61], growth and drought [84] in several forest tree species [85]. In our study, we found that the homozygous of the minor allele (CC) for ABC gene PSME_40921 was associated with increased root to shoot ratio-RTSH (slower growth); and usually occurs at mountainous regions (elevation > 1000 masl) with more than 30 chilling degree-days in autumn. 

## 5. Conclusions and Predictions in the Face of Climate Change

Due to the rapid nature of climate change and the slow nature of plant migrations, proactive strategies should be employed by forest managers to mitigate potential damages resulting from the increased frequency of extreme cold and drought events [86]. As global climate warms at unprecedented rates, species’ distributions are expected to change exposing populations to new environmental conditions [87]. Climate prediction models suggest current climate change could reduce the resilience of coastal Douglas-fir on the warmer margins of its range [21], which will translate in a reduction in fitness and therefore lower wood productivity. Populations in colder areas might also suffer from an abrupt growth decline as a consequence of late spring frosts (more common under warming climates), as previously observed in Canadian populations of *Picea mariana* [88]. Moreover, populations of Douglas-fir that are isolated on mountains, especially southern populations of the intermountain variety, should be of particular interest due to their higher risk of losing genetic diversity [89]. Our study indicated that these mountainous regions harbor low-frequency alleles that have very important roles in cold adaptation in coastal Douglas-fir. As predictions of future climate variation improve, so should our understanding of the role of phenotypic variation in the potential for adaptation to changing environments.

## Figures and Tables

**Table 1:** Genetic architecture of cold-related phenotypic traits in coastal Douglas-fir.

Trait	PVE	PGE	SNPs	BSLMM	MLM
trait1	0.75 ± 0.17	0.52 ± 0.16	3 ± 2	23	106
trait2	0.88 ± 0.13	0.24 ± 0.2	78 ± 71	1617	0
BB2	0.65 ± 0.19	0.38 ± 0.19	3 ± 2	15	25
BS1	0.6 ± 0.17	0.56 ± 0.18	3 ± 2	11	70
BS2	0.68 ± 0.21	0.13 ± 0.16	34 ± 40	52	0
DIAM	0.8 ± 0.16	0.37 ± 0.14	2 ± 1	11	75
EMEAN	0.86 ± 0.16	0.21 ± 0.1	3 ± 4	18	54
EMSTD	0.47 ± 0.2	0.45 ± 0.21	3 ± 2	8	48
FLUSH	0.39 ± 0.25	0.2 ± 0.24	17 ± 24	18	1
FLUSHLG	0.26 ± 0.19	0.27 ± 0.27	25 ± 38	26	0
HT1	0.64 ± 0.2	0.41 ± 0.18	2 ± 1	6	46
HT2	0.54 ± 0.16	0.64 ± 0.19	2 ± 1	14	77
HTINC	0.52 ± 0.19	0.5 ± 0.22	3 ± 2	15	54
RTLG	0.68 ± 0.23	0.34 ± 0.2	10 ± 18	34	30
RTSH	0.48 ± 0.14	0.71 ± 0.18	2 ± 2	14	115
RTWT	0.76 ± 0.21	0.22 ± 0.16	3 ± 5	23	20
SHWT	0.66 ± 0.22	0.33 ± 0.2	3 ± 6	18	29
TAPER	0.69 ± 0.23	0.23 ± 0.21	38 ± 54	188	10
TOTWT	0.69 ± 0.21	0.31 ± 0.18	2 ± 2	22	30
budcold	0.65 ± 0.19	0.29 ± 0.25	99 ± 84	2453	1
ndlcold	0.93 ± 0.09	0.42 ± 0.24	96 ± 78	2857	2
stmcold	0.88 ± 0.13	0.15 ± 0.13	11 ± 16	34	4
SDWT	0.82 ± 0.19	0.13 ± 0.15	23 ± 35	58	2

Results from the multivariate Bayesian Sparse Linear Mixed Model (BSLMM) in GEMMA and its comparison with univariate Mixed Linear Model (MLM) in TASSEL. Variables are phenotypic trait (Trait), proportion of variance explained (PVE), proportion of variance explained by sparse effects (PGE), posterior estimate of number of SNPs with major effect and the standard deviation across runs after burn-in (SNPs), number of SNPs with significant associations identified by GEMMA, and number of SNPs with significant associations identified by TASSEL. Traits included: Trait1 (First canonical variable for traits), Trait2 (Second canonical variable for traits), BB2 (Bud burst in year 2), BS1 (Bud set in year 1), BS2 (Bud set in year 2), DIAM (Diameter after year 2), EMEAN (Rate of emergence), EMSTD (Standard deviation of emergence rate), FLUSH (Propensity to second flush), FLUSHLG (Length of second flush), HT1 (Height after year 1), HT2 (Height after year 2), HTINC (Height increment between years 1 and 2), RTLG (Root Length), RTSH (Root: shoot ratio), RTWT (Root weight), SHWT (Shoot weight), TAPER (Taper), TOTWT (Total weight), budcold (Cold damage of buds), ndlcold (Cold damage of needles), stmcold (Cold damage of stems) and SDWT (Weight of 100 seeds).

**Table 2:** Functional characterization of genes associated with cold adaptation in coastal Douglas-fir.

Gene	NCBI Accession	S	O	Trait	Env.	Processes/Pathways	Functional Annotation
PSME_32494	XP_020083319.1	1	N	cold	TD	Pentose phosphate pathway	glucose-6-phosphate 1-dehydrogenase 4
PSME_35569	XP_011080970.1	1	N	cold	SHM	Cell wall pectin biosynthesis	xyloglucan-specific galacturonosyltransferase 1
PSME_01956	XP_023919718.1	1	N	cold	bFFP	Starch and sucrose metab.	neutral trehalase-like
PSME_04717	XP_023911511.1	2	Y	grow cold	TD, PC1	CH and aminoacid metab.	4-aminobutyrate aminotransferase
PSME_39868	XP_020187038.1	1	N	cold	PAS, TD	Glycolysis, fatty acid degradation, tyrosine metab.	alcohol dehydrogenase-like 4
PSME_34754	XP_011622948.1	1	Y	grow	TD	Glycolisis	plastidial pyruvate kinase 4
PSME_37622	XP_023873676.1	1	N	cold	Lat	Glycolysis, phosphorylation of fructose-6-phosphate	ATP-dependent 6-phosphofructokinase 5
PSME_46816	MA_72344g0010	1	N	cold	TD	sugar transporter	Bidirectional sugar transporter SWEET3-like
PSME_47335	XP_006858646.2	1	N	cold	TD	sugar transporter	CMP-sialic acid transporter 5
PSME_39947	XP_014501229.1	1	N	grow phe	DD_0, TD	Fructose and mannose metabolism	mannan endo-1,4-β-mannosidase 7-like
PSME_39362	RVW55203.1	4	N	grow cold	EXT, Eref, TD	cellulose metabolism	cellulose synthase
PSME_40047	XP_020534550.1	1	N	cold	TD	cellulose metabolism	β-glucosidase 18
PSME_20810	MA_121907g0010	1	N	cold	TD	CH and photosynthesis	plastocyanin-like
PSME_32128	XP_020532303.1	1	N	phe	MCMT	Glycerophospholipid metab.	lysophospholipid acyltransferase LPEAT2
PSME_23694	XP_011100368.1	1	N	cold phe	TD	arginine, proline, β-alanine, pantothenate and CoA	polyamine oxidase 2
PSME_30851	XP_006836334.1	1	Y	grow cold	DD5_at	arginine, proline, β-alanine, pantothenate and CoA	polyamine oxidase 2
PSME_40791	XP_004289831.1	1	N	grow cold	DD_0_at	Cysteine and methionine metabolism	5’-methylthioadenosine/S-adenosylhomocysteine nucleosidase
PSME_01345	XP_023540379.1	1	N	grow	TD	RNA metabolism	DEAD-box ATP-dependent RNA helicase 57
PSME_39361	XP_021895115.1	3	N	grow cold	PAS, TD, DD_0	Terpenoid backbone biosynthesis	1-deoxy-D-xylulose 5-phosphate reductoisomerase
PSME_35875	XP_023520455.1	1	N	grow cold phe	TD	Ubiquinone and other terpenoid-quinone biosynthesis	NAD(P)H dehydrogenase (quinone) FQR1
PSME_03065	XP_023925599.1	1	N	phe	PAS	Steroid and diterpenes biosynthesis	cytochrome P450
PSME_13751	XP_007209932.1	1	N	cold	MWMT, TD	Steroid biosynthesis	geraniol 8-hydroxylase, cytochrome P450
PSME_06220	XP_018684598.1	1	Y	cold	PAS	Glycosylation (terpenes and others biosynthesis)	UDP-rhamnose:rhamnosyltransferase 1
PSME_42889	XP_021819748.1	1	N	cold phe	PC1	Porphyrin and chlorophyll metabolism	phytochromobilin:ferredoxin oxidoreductase
PSME_41367	XP_011080788.1	1	N	cold	PAS	vitamin B6 metabolism	pyridoxal 5’-phosphate synthase subunit PDX1
PSME_28922	XP_007202107.1	1	N	grow	DD_0, CMD	flavonoid biosynthesis	naringenin,2-oxoglutarate 3-dioxygenase
PSME_40932	XP_023531135.1	2	N	grow cold phe	DD_0, MSP, TD	growth and development	protein COBRA-like
PSME_04771	XP_006433957.1	1	N	grow cold phe	DD_0, EXT, TD	growth and development	EXORDIUM-like 2
PSME_02459	XP_023541193.1	1	N	cold phe	TD	growth and development	EXORDIUM-like 3
PSME_32867	XP_020102207.1	1	N	grow phe	DD_0, TD	growth and development	NAC domain-containing protein 68-like
PSME_31370	MA_101849g0010	1	N	grow	TD	reproductive development	NAC domain-containing 35-like (PF02365)
PSME_17369	XP_016710129.1	1	N	grow phe	DD_0, TD	growth and development	PREDICTED: LOB domain-containing protein 19
PSME_47800	XP_023516539.1	1	N	grow phe	DD_0, TD	growth and development	probable inactive purple acid phosphatase 27
PSME_40261	PSS23947.1	1	N	grow phe	DD_0_at, PC1	growth and development	Purple acid phosphatase
PSME_30046	BBC78345.1	1	N	grow	DD_0, Eref	reproductive development	NEEDLY-like protein
PSME_37515	XP_010242469.1	1	N	phe	TD	reproductive development	LETM1 and EF-hand domain-containing protein 1
PSME_47186	XP_011627569.1	1	N	grow	DD_0_at, PAS	growth	methionine aminopeptidase 1B
PSME_27219	XP_012838463.1	1	N	grow cold phe	DD_0, PC1, TD	cell cycle control	cyclin-dependent kinase C-2-like isoform X2
PSME_38027	XP_031497943.1	1	N	grow cold	TD	cell cycle control	GAMETE EXPRESSED 1
PSME_51103	XP_022153266.1	1	N	grow phe	DD_0, Lat, TD	Transcription regulation	CASP isoform X2
PSME_30616	XP_001754344.1	1	N	grow phe	PC1, TD	Transcription regulation	TATA-box binding
PSME_18347	MA_8626g0010	1	N	phe	EXT	Transcription regulation	Tannin-related R2R3 MYB transcription
PSME_01485	MA_4929994g0010	1	N	cold	PC1	Transcription regulation	AP2 domain transcription factor
PSME_34977	MA_2589g0010	1	N	cold	DD_0_at	Transcription regulation	Transcription termination factor
PSME_46703	KHN01498.1	1	N	grow	TD	Transcription, splicing factor SF1	KH Domain-containing protein
PSME_37207	MA_9457778g0010	1	N	grow cold phe	DD_0_at, TD	antioxidative defense	peroxidase 5 (PF00141)
PSME_46413	XP_021618646.1	1	N	grow phe	PAS, TD	antioxidative defense	peroxidase 4
PSME_40921	MA_629244g0010	1	N	grow	DD_0_at	Biotic stress	ABC transporter C family member 14-like
PSME_16294	XP_021726759.1	1	N	grow	TD	Biotic stress, plant-pathogen interaction	protein SGT1 homolog
PSME_47942	MA_35233g0010	1	N	grow phe	TD	Salt stress	cysteine rich repeat secretory 5 (PF01657)
PSME_32719	XP_020268797.1	1	N	grow	TD	abiotic stress	17.8 kDa class I heat shock protein-like
PSME_47504	ADB97926.1	1	N	grow phe	DD_0, AHM, TD	cold stress	thaumatin-like L2
PSME_33611	XP_010920821.1	1	N	grow cold	TD	biotic stress	protein PMR5
PSME_02881	OVA07401.1	1	Y	grow phe	PC1, TD	abiotic stress	Leucine-rich repeat
PSME_03186	MA_52212g0010	1	N	cold phe	PAS	stress and calcium signaling	Probable calcium-binding CML25
PSME_09147	XP_021984819.1	1	N	grow cold phe	DD_0_at, TD	biotic stress, signal transduction	serine/threonine-protein kinase AFC1-like
PSME_32617	XP_011077233.1	1	N	grow cold phe	PC1, TD	biotic stress, signal transduction	phosphoenolpyruvate carboxylase kinase 2
PSME_42855	XP_021297375.1	1	N	cold	PAS, TD	biotic stress, signal transduction	protein kinase and PP2C-like domain-protein
PSME_08981	XP_020099349.1	1	N	grow cold	EXT	stress, vesicular transport	ras-related protein Rab7
PSME_47475	XP_021275390.1	1	Y	phe	TD	stress, signal transduction	lanthionine synthetase component C (lanC)-like protein GCL1
PSME_05154	XP_007218270.1	1	Y	grow	SHM	Biotic stress	carboxylesterase 15
PSME_01336	XP_010269100.1	1	N	grow phe	DD_0_at, TD	Biotic stress	carboxylesterase 2
PSME_34054	MA_15220g0010	1	Y	grow	TD	Biotic stress	Metallothiol transferase
PSME_00292	XP_023900051.1	1	N	grow phe	DD_0, MSP, TD	Ubiquitin mediated proteolisis	ubiquitin-40S ribosomal protein S27a
PSME_03290	XP_023547970.1	1	N	phe	PAS	Ubiquitin mediated proteolisis	U-box domain-containing protein 26
PSME_02609	XP_016487911.1	1	N	grow cold phe	Long, TD	Ubiquitin mediated proteolisis	cullin-1-like
PSME_01721	MA_83109g0010	1	Y	grow	DD_0	Ubiquitin mediated proteolisis	cullin-1 like
PSME_04517	XP_023538234.1	1	N	cold phe	SHM	Ubiquitin mediated proteolisis	SKP1-like protein 1B
PSME_37900	MA_10432666g0010	1	Y	grow cold	TD	Ubiquitin mediated proteolisis	U11 U12 small nuclear ribonucleo 25 kDa
PSME_15528	XP_010250248.1	1	N	cold	PC3	Ubiquitin mediated proteolisis	ubiquitin carboxyl-terminal hydrolase 22
PSME_02072	XP_024392985.1	1	N	grow	TD	Ubiquitin mediated proteolisis	E3 ubiquitin protein ligase RING1-like
PSME_30516	AAX92710.1	1	N	grow	PAS	Ubiquitin mediated proteolisis	SCF ubiquitin ligase
PSME_48521	XP_022751818.1	1	N	cold	TD	stress, signal transduction	histidine kinase 5-like
PSME_27170	XP_021677291.1	1	N	grow phe	PAS	stress, signalling and cellular processes	pollen-specific protein SF21-like isoform X2
PSME_30964	MA_279935g0010	1	N	grow	DD_0_at, TD	Methylation and growth	Histone-lysine N-methyltransferase family member SUVH9
PSME_15751	XP_012079811.1	1	N	cold	TD	Methylation and growth	putative methyltransferase DDB_G0268948
PSME_40578	KAF5183317.1	1	N	cold	Elev	Merthylation	23S rRNA (uracil(1939)-C(5))-methyltransferase RlmD
PSME_32636	XP_021691879.1	1	N	grow	PC2	translation	50S ribosomal protein L35, chloroplastic-like
PSME_02851	XP_013468200.1	1	N	phe	PAS, TD	translation	60S ribosomal L12-like
PSME_36577	XP_020101722.1	1	N	phe	MAP	translation	probable GTP-binding protein OBGM, mitochondrial
PSME_46659	RWR94859.1	1	N	cold	PAS, TD	translation	eukaryotic translation initiation factor 5B
PSME_31058	XP_020522223.1	1	N	grow	MSP, TD	DNA replication and repair	crossover junction endonuclease MUS81
PSME_01087	XP_004229823.1	1	N	grow phe	PAS, TD	DNA replication and repair	AT-hook motif nuclear-localized protein 22
PSME_41853	XP_010277286.1	1	N	grow	PC1, TD	post-translational protein modification	PREDICTED: protein S-acyltransferase 11

Variables include: Gene ID in the Douglas-fir genome version Psme v1.0; NCBI accession; Number of SNP markers (S); Results of PCAdapt outlier test (O); GWAS associated traits divided in three categories: growth and emergence (grow), phenology (phe) and cold hardiness (cold); GEA associated environmental variables; Processes or Pathways, and Functional Annotation. SNPs matching transcripts and genes with unknown function were not included. Key to environmental variables can be found in Appendix A, Table A2. Accession IDs starting with “MA” can be found at congenie.org. CH = carbohydrate.

## Data Availability

The data presented in this study are available in Appendix A and Supplementary Materials.

## Supplementary Materials

The following are available online at https://www.mdpi.com/2073-4425/12/1/110/s1, Table S1: Results of the outlier analysis results, Table S2: Results of the GWAS analyses implemented in TASSEL, Table S3: Results of the GWAS analyses implemented in GEMMA, Table S4: Results of the GEA analyses implemented in TASSEL, Table S5: Results of the GEA analyses implemented in Bayenv, Table S6: Results of the multivariate GEA analyses using Redundancy Analysis (RDA), Table S7: Candidate SNPs for cold adaptation in Douglas-fir.

## Appendix A

**Table A1:** Key to phenotypic traits. Variables included are: Biological process (Bio Process), Trait abbreviation (Trait ID), full trait name (Trait description), and units of measurement (Unit).

Bio Process	Trait ID	Trait Description	Unit
Several	Trait1	First canonical variate for traits	--
Several	Trait2	Second canonical variate for traits	--
Phenology	BB2	Budburst in year 2	days since Jan 1
Phenology	BS1	Budset in year 1	days since Jan 1
Phenology	BS2	Budset in year 2	days since Jan 1
Growth	DIAM	Diameter after year 2	mm
Emergence	EMEAN	Rate of emergence	probits/day
Emergence	EMSTD	Standard deviation of emergence rate	probits/day
Emergence	FLUSH	Propensity to second flush	proportion
Emergence	FLUSHLG	Length of second flush	cm
Growth	HT1	Height after year 1	cm
Growth	HT2	Height after year 2	cm
Growth	HTINC	Height increment between years 1 & 2	cm
Growth	RTLG	Root length	cm
Growth	RTSH	Root:shoot ratio	g/g
Growth	RTWT	Root weight	g
Growth	SHWT	Shoot weight	g
Growth	SDWT	Weight of 100 seeds	g
Growth	TAPER	Taper	mm/cm
Growth	TOTWT	Total weight	g
Cold hardiness	budcold	Cold damage of buds	%/10
Cold hardiness	ndlcold	Cold damage of needles	%/10
Cold hardiness	stmcold	Cold damage of stems	%/10

**Table A2:** Key to environmental traits. Variables included are: Variable name abbreviation (Variable ID), full variable name (Variable name), units of measurements (Unit), and a description of the variable (Variable Description).

Variable ID	Variable Name	Unit	Variable Description
Latitude	Latitude	degrees	From GIS after mapping parents.
Longitude	Longitude	degrees	From GIS after mapping parents.
Elevation	Elevation	m	From DEM after mapping parents.
MAT	Mean annual temperature	degrees C	Mean annual temperature
MWMT	Mean warmest month temperature	degrees C	Mean warmest month temperature
MCMT	Mean coldest month temperature	degrees C	Mean coldest month temperature
TD	Temperature difference between MWMT and MCMT, or continentality	degrees C	Temperature difference between MWMT and MCMT, or continentality
MAP	Mean annual precipitation	mm	Mean annual precipitation
MSP	May to September precipitation	mm	May to September precipitation
AHM	Annual heat-moisture index		(MAT+1-)/(MAP/1000)
SHM	Summer heat-moisture index		(MWMT)/(MSP/1000)
DD_0	Degree-days below zero	days	chilling degree-days
DD_0_sp	Degree-days below zero in spring	days	chilling degree-days in spring
DD_0_at	Degree-days below zero in autumn	days	chilling degree-days in autumn
DD5	Degree-days above 5 °C	degrees C	growing degree-days
DD5_sp	Degree-days above 5 °C in spring	degrees C	growing degree-days in spring
DD5_at	Degree-days above 5 °C in autumn	degrees C	growing degree-days in autumn
NFFD	Number of frost-free days	days	Number of frost-free days
FFP	Frost-free period	days	Frost-free period
bFFP	The day of the year FFP begins	day of year	The day of the year FFP begins
eFFP	The day of the year on which FFP ends	day of year	The day of the year on which FFP ends
PAS	Precipitation as snow	mm	Precipitation as snow
EMT	Extreme minimum temperature	degrees C	Lowest temperature over 30 years
EXT	Extreme maximum temperature	degrees C	Highest temperature over 30 years
Eref	Hargreaves reference evaporation	mm	Hargreaves reference evaporation
CMD	Hargreaves climatic moisture deficit	mm	Hargreaves climatic moisture deficit
PC1	Principal component 1		First principal component of climate variables
PC2	Principal component 2		Second principal component of climate variables
PC3	Principal component 3		Third principal component of climate variables
